# Biocontrol in built environments to reduce pathogen exposure and infection risk

**DOI:** 10.1093/ismejo/wrad024

**Published:** 2024-01-10

**Authors:** Neil R Gottel, Megan S Hill, Maxwell J Neal, Sarah M Allard, Karsten Zengler, Jack A Gilbert

**Affiliations:** Center for Marine Biotechnology and Biomedicine, Scripps Institution of Oceanography, University of California San Diego, La Jolla, CA 92037, United States; Center for Marine Biotechnology and Biomedicine, Scripps Institution of Oceanography, University of California San Diego, La Jolla, CA 92037, United States; Department of Pediatrics, School of Medicine, University of California San Diego, La Jolla, CA 92093, United States; Department of Bioengineering, University of California San Diego, La Jolla, CA 92093, United States; Center for Marine Biotechnology and Biomedicine, Scripps Institution of Oceanography, University of California San Diego, La Jolla, CA 92037, United States; Department of Pediatrics, School of Medicine, University of California San Diego, La Jolla, CA 92093, United States; Department of Pediatrics, School of Medicine, University of California San Diego, La Jolla, CA 92093, United States; Department of Bioengineering, University of California San Diego, La Jolla, CA 92093, United States; Center for Microbiome Innovation, University of California San Diego, La Jolla, CA 92093, United States; Center for Marine Biotechnology and Biomedicine, Scripps Institution of Oceanography, University of California San Diego, La Jolla, CA 92037, United States; Department of Pediatrics, School of Medicine, University of California San Diego, La Jolla, CA 92093, United States; Center for Microbiome Innovation, University of California San Diego, La Jolla, CA 92093, United States

**Keywords:** indoor microbiome, biocontrol, probiotic cleaning, bacillus, antibiotic resistance, built environment, AMR, metabolic modelling

## Abstract

The microbiome of the built environment comprises bacterial, archaeal, fungal, and viral communities associated with human-made structures. Even though most of these microbes are benign, antibiotic-resistant pathogens can colonize and emerge indoors, creating infection risk through surface transmission or inhalation. Several studies have catalogued the microbial composition and ecology in different built environment types. These have informed *in vitro* studies that seek to replicate the physicochemical features that promote pathogenic survival and transmission, ultimately facilitating the development and validation of intervention techniques used to reduce pathogen accumulation. Such interventions include using *Bacillus*-based cleaning products on surfaces or integrating bacilli into printable materials. Though this work is in its infancy, early research suggests the potential to use microbial biocontrol to reduce hospital- and home-acquired multidrug-resistant infections. Although these techniques hold promise, there is an urgent need to better understand the microbial ecology of built environments and to determine how these biocontrol solutions alter species interactions. This review covers our current understanding of microbial ecology of the built environment and proposes strategies to translate that knowledge into effective biocontrol of antibiotic-resistant pathogens.

## Introduction

Cleaning and disinfection practices in homes and hospitals to combat bacteria, fungi, and viruses have a long history. Ancient civilizations used various disinfection methods to remove “pestilence,” from the rudimentary practices of boiling and sunlight exposure to the use of natural substances like vinegar and sulphur [1]. However, it was not until the 19th century that the age of modern infection control began to take shape with targeted cleaning practices. For example, Ignaz Semmelweis introduced hand disinfection in hospitals [2], and Florence Nightingale introduced best practices to decrease infection rates and improve patient healing [3]. In the following decades, the use of carbolic acid by Joseph Lister for surgical instrument sterilization, in conjunction with other antiseptic methods, further enhanced pathogen control in clinical settings [4]. By the early-to-mid 20th century, the discovery of antibiotics and the development of chemical disinfectants, such as phenol and bleach, provided a newfound defence against infectious agents [5]. Though antibiotics have been highly successful and saved many lives, the overuse of these drugs has led to the emergence of antibiotic-resistant bacteria, such as methicillin-resistant *Staphylococcus aureus* (MRSA) and vancomycin-resistant *Enterococcus*, making traditional antibiotics less effective [6]. Additionally, pathogens are evolving resistance to biocidal compounds used in traditional cleaning solutions [7]. However, chemical treatments such as phenol and bleach remain effective despite providing only temporary removal of pathogens on surfaces.

To reduce the risks associated with various disinfection cleaning agents, hospitals now more frequently use a variety of cleaning strategies for disinfection, including advanced technologies like exposure to ultraviolet (UV) light and hydrogen peroxide vapour [8]. Despite these powerful treatments, outbreaks of *Clostridioides difficile* [9] and other antimicrobial-resistant (AMR) organisms [10-14] have continued to rise within healthcare settings, highlighting the enduring challenge of simultaneously disinfecting an area while avoiding selection for traits that increase pathogenic potential. Reducing the spread of AMR pathogens is a crucial priority for global human health. In 2019, 1.27 million deaths were directly caused by AMR pathogens globally [15], and they are projected to cause an additional 10 million deaths annually by 2050 [16]. We must therefore bolster our defences against persistent and emerging pathogens, safeguarding global health and well-being amidst the evolving landscape of microbial threats [17].

The inability of current hospital disinfection methods to fully combat these challenges emphasizes the crucial need for continuous research, development, and adaptation of infection control methods. Although traditional cleaning methods have helped to reduce the emergence and spread of antibiotic-resistant bacteria and viruses in the built environment, using microbes sourced from different environments to inhibit pathogen survival and transmission is an intriguing new tool that is posed to further improve the health of people indoors. Reintroducing specific microbes into buildings can potentially revolutionize how we prevent the emergence and persistence of unwanted microbes. However, deploying what could be called a microbial biocontrol product is hampered by a lack of basic understanding of the mechanisms of action for this ecological strategy to control disease. Despite this knowledge gap, cleaning products containing bacteria that demonstrate antimicrobial activity have been developed [18] and are already publicly available for purchase. *Bacillus* spores are often used because many species are generally recognized as safe, nonpathogenic, produce a wide variety of antimicrobial compounds [19], and remain viable over long periods of time [20]. Though it is unknown how effectively these spores germinate on dry, nutrient-depleted surfaces in built environments, it is important to determine why they are found to be associated with a reduction in the abundance of hospital-associated pathogens [21-24]. It is unclear whether there is an actual competitive exclusion or inhibition effect or if the spores occupy niches on surfaces, thereby denying this space to pathogens. Alternatively, the spores may mask any detectable signal of the pathogens, as determined via DNA sequencing methods. If the latter is true, this could mean that the pathogens remain viable and can cause infection. Answering this open question and moving the field towards improved public health objectives will require using a diverse suite of experimental and analytical approaches, including techniques that have been so far underutilized in built environment research.

In the last decade, there have been critical innovations in the methods, study approaches, and experimental designs used to characterize diversity, survival, distribution, transmission, and health risk of microbes found in our buildings. A rapid explosion in observational studies over the last 15 years has provided a baseline analysis of the microbial diversity in built environments. High-throughput sequencing of bacterial 16S rRNA and fungal internal transcribed spacer (ITS) genes has demonstrated that microbes colonize and spread throughout buildings in predictable ways [25-30]. In addition, shotgun metagenomic sequencing has been used to characterize the spread of AMR genes [31] and to quantify the relative abundance of microbial taxa across surfaces and on the skin of indoor environment occupants [32]. Also, quantitative polymerase chain reaction (qPCR) has been employed to determine the absolute abundance of specific AMR genes [33, 34] or species of interest [35] in a diverse set of environments such as soil, water, air, faeces, and sediments. Further, targeted and untargeted metabolomics have been employed to characterize the distribution of chemicals around built spaces and infer the metabolic ecology of species interactions in experimental systems and real-world environments [36-38]. These methods can be enhanced using specialized DNA extraction techniques and internal standards to compensate for the low biomass typically recovered in built environment samples [32, 39, 40].

Investigations in real-world built spaces have led to numerous new hypotheses regarding microbial survival and transmission, and the metabolic interactions that underpin these properties. Robustly testing these hypotheses has required innovations in experimental laboratory-based studies. Despite laboratories being built spaces themselves, recreating the inherent ecological dynamics that define the indoor microbiome has proven difficult and has required a reimagining of traditional microbiology techniques. Microbes in the built environment are generally assumed to be starving, dying, or dead [41]. We are, therefore, attempting to study life on the edge of survival. Many microorganisms are unable to survive on surfaces, but some pathogens have demonstrated the ability to survive on surfaces common to the built environment. For example, MRSA shows robust viability on surfaces such as vinyl and plastic over days [42]. Traditional microbiology cultivates microbes using media optimized over decades of experimentation to encourage their growth. Such conditions are unlikely to replicate microbial interactions in the built environment. So, it is necessary to use techniques that more closely reproduce the harsh conditions of most built environment surfaces to more accurately examine how microbes survive, grow, and interact in this environment.

Observational studies and laboratory investigations inform intervention studies in ways that help determine the potential benefit of removing species of concern or introducing potentially beneficial microbes, both for the integrity of the building structure and the health of the occupants. Intervention studies have been performed in mock built environment microcosms, as well as in full-scale field trials, and are now elucidating the ecological dynamics of these environments. Unfortunately, due to the cost and added risk of conducting an intervention within buildings such as hospitals, only a few full-scale tests of these intervention techniques have been completed. This review will discuss recent advances in built environment microbiome research, with a focus on strategies related to the understanding and translation of biocontrol practices used to reduce antibiotic-resistant pathogens on surfaces in real-world clinical settings.

## Techniques for investigating the microbiome of the built environment

Observational studies have investigated microbial dynamics in the built environment, detecting broad trends in the microbial composition and indoor ecological dynamics. For example, these studies describe how the diversity, composition, and functional potential of communities change over time and how occupants and building operations influence these interactions. Many built environments have been explored in this way, including workplaces [43-45], homes [46-49], and public transportation [50, 51]. Of critical importance to understanding the interaction between surface-associated microbial ecology and pathogenic activity, hospital environments have been well characterized [25, 26, 52], providing some of the most compelling results to justify further intervention studies. For example, one investigation demonstrated that surfaces in a newly built hospital were inoculated with microbes that closely resembled the outside environment [25]. However, when that hospital became operational and therefore densely populated with patients and healthcare workers, the surface-associated microbiome began to resemble that of the occupants’ skin and respiratory tract. In addition, following the start of operational activity, hospital custodians cleaned surfaces with defined periodic frequency, which also influences microbial dynamics [25]. Similar trends have been observed in studies that focused on closed hospital wards that were renovated and reopened for use [27]. Using metagenomic reconstruction of bacterial genomes in hospitals over dense time series has demonstrated that bacteria in the hospital environment have increased selection pressure for the acquisition and accumulation of antimicrobial resistance genes [25]. Although the mechanism of action has not been elucidated, bacterial adaptation and resistance to disinfectants (such as those used to clean the hospital) is well known [53], and we hypothesize that genetic adaptation to survival against such disinfection may also be concomitant with other genetic survival strategies, such as antimicrobial resistance. However, further research is needed to uncover the explicit relationship between these phenomena.

Over the last 15 years, observational studies have generally applied relatively affordable microbiome analysis techniques, such as 16S rRNA or ITS gene sequencing, to characterize the diversity and taxonomic composition of bacterial and fungal communities, and qPCR of targeted genes to determine absolute abundances of microbial community members [54-58]. Although approaches such as metagenomics, metatranscriptomics, metabolomics, and application of qPCR to quantify a variety of genes and microbial activity (e.g. the abundance of antibiotic resistance genes) can dramatically increase experimental costs, they can significantly improve our understanding of microbial interactions in these settings [59-61]. These methods can be implemented to identify patterns of microbial metabolism in complex systems [62], or when observed over time, these data can reveal temporal dynamics. However, the implementation of these other ‘omics approaches is challenging due to the low microbial biomass found on surfaces in buildings, especially where resources such as water, carbon, nitrogen, and other essential nutrients to sustain life are limited and there is frequent cleaning [41, 45]. Additionally, although observing spatial or temporal trends in microbiome composition or metabolism is useful, it is hard to identify the features that influence these dynamics due to our inability to control for every factor in a real-world environment. Targeted interventions or manipulations of the environment and the subsequent measurement of the magnitude of effect are needed to better understand how to optimize beneficial change. Even in an observational study such as the Hospital Microbiome Project [25], where we can observe a pseudo-intervention, in this case a change in occupancy status in real-time, it is not possible to explicitly track the movement of individual microbes or infer microbial metabolic interactions and competitive dynamics. However, it is possible to predict how microbes interact with each other and model their behaviour in these environments using metabolic models [63].

### Modelling microbial metabolism

To improve our understanding of microbial activity and ecology in these extreme environments, it is necessary to fundamentally understand their metabolic potential. Genome-scale metabolic models (GSMMs) are mathematical models of metabolic networks reconstructed from the organism’s annotated genome [63, 64]. Genome-scale metabolic modelling can help facilitate a clear understanding of the mechanisms that microbes deploy to survive, germinate, and compete for resources. These models predict growth rates in a simulated medium and determine what reaction rates are needed to support growth. Pairs or larger groups of models can be connected to determine which metabolites are being readily exchanged (commensally or symbiotically) and which limited nutrients drive competition between the organisms [65, 66]. These models can make predictions for hundreds of conditions within seconds, allowing for the rapid identification of important nutrients, uptakes, and secretions, thereby rationally informing the design of subsequent experiments. In an iterative process, experimental data can be incorporated into GSMMs, contextualizing the data and further refining their predictions. Transcriptomics can also be used to detect reactions associated with low-transcription genes, increasing the specificity of the model to the environment of interest [67]. The refined model then yields insights into the biological relevance of transcriptional changes by identifying how they affect the expression of larger metabolic pathways. These models can also facilitate the analysis of multiomics data, by integrating them into an interpretable scaffold, which can then be used to predict how changes in the genome or to the environment influence survival or competitive outcomes. Using these modelling techniques on samples from the International Space Station, an extreme built environment revealed beneficial interactions between the pathogen *Klebsiella pneumoniae* and bacterial species of the genus *Pantoea* and the family *Enterobacteriaceae* [68]. The model also predicted *K. pneumoniae* is parasitic towards the fungal genus *Aspergillus*, which was confirmed experimentally via co-culture [68]. Predicting microbial interactions in built environments via modelling is a powerful technique to inform efficacious biocontrol strategies by identifying novel species interactions and predicting their differential effect in a range of scenarios. This information can be generated based solely on ‘omics data and subsequently confirmed in laboratory studies.

### Interpreting metabolic ecology

Studies in controlled laboratory settings typically, but not always [69], use microcosms that contain small pieces of construction materials relevant to the built environment of interest, and conditions (e.g. humidity and temperature) are varied to determine whether they have an effect on microbial activity and survival. To mimic realistic microbial community interactions and successional dynamics, studies have seeded surface materials with microbes by leaving them exposed within built environments prior to initiating challenge studies. In a study of microbial metabolic dynamics [60], coupons of oriented strand board, medium-density fibreboard, regular gypsum wallboard, and mould-resistant gypsum were naturally inoculated by passive colonization in homes and in a laboratory. Following this seeding, the coupons were either soaked in water to simulate a water leak or kept dry and then all coupons were incubated for an additional 30 days within a high humidity (~94%) chamber. Swabs of the coupons were collected every 5 days and analysed with 16S/ITS rRNA gene amplicon sequencing and metabolomics to determine how these communities changed taxonomically and metabolically in response to wetting. Wetted coupons were dominated by the bacterial genera *Bacillus*, *Erwinia*, and *Pseudomonas* and the fungal genera *Eurotium* and *Penicillium*. When coupons had been wetted, *Bacillus* and *Pseudomonas* species were almost always negatively correlated in relative abundance, suggesting competitive exclusion. Even though *Bacillus* species are known to produce antifungal compounds [70-72], only the mould-resistant gypsum was dominated by *Bacillus*. The antibacterial compounds nigragillin and fumigaclavine C were found in high abundance. They were positively correlated with the presence of *Aspergillus* and negatively correlated with the abundance of *Bacillus* and *Pseudomonas* [60]. These results suggest that the presence of *Bacillus* is not sufficient to prevent fungal growth on built environment surfaces. Although they did not purposefully inoculate surfaces with *Bacillus*, this study suggests that this genus did proliferate when coupons were wetted, but it only dominated on mould-resistant gypsum, which inherently inhibits fungal growth, and as such, the naturally occurring *Bacillus* species may not have antifungal activity.

Semi-*in vitro* investigations are effective at discovering novel microbial interactions, but ecological dynamics can also be inferred from applying multiomic techniques to controlled building environments. For example, one study characterized the chemical and microbial compositions of two frequently wet surfaces in a residential setting, specifically the kitchen sink and bathroom shower [73]. This study used a combination of culture-dependent and independent techniques, including transcriptional analysis of the 16S rRNA gene to determine which bacteria were active at the time of sampling, and assessment of both volatile and soluble chemicals to explore the links between the observed microbiota and chemical exudates. This study showed that microbes play a critical role in structuring the chemical profiles of surfaces in built environments, particularly in kitchen sinks and shower stalls. The microbial volatile organic compounds (mVOCs) were predominantly associated with fatty acid processing, and the composition of these mVOCs appeared more stable than that of the microbial communities themselves, which showed variations in response to changing environmental conditions. A second example demonstrated microbial colonization, succession, and viability in a tightly controlled restroom environment [61]. This study focused on the ecological succession and viability of human-associated microbiota on restroom surfaces, including floors, toilet seats, and soap dispensers. The study demonstrated that a late-successional microbial community develops on restroom surfaces within 5 to 8 hours of decontamination. This community showed remarkable stability over weeks to months, indicating a quick establishment and persistence of specific microbial assemblages. The authors also showed that faecal taxa, particularly those able to enter a dormant phase, can persist for extended periods on restroom surfaces. This insight into microbial survival strategies in dry and nutrient-poor environments of built environments is crucial for understanding disease transmission, ecology, and environmental health. This study also found a significant positive correlation between bacterial and viral abundances, but with an unexpectedly low virus-to-bacterium ratio of nearly 1:1. This suggests that many bacteria on restroom surfaces are in a dormant state, influencing the dynamics of phages in these environments. Understanding microbial ecological dynamics in these systems is essential if we are able to control the survival, emergence, and persistence of pathogens. Next, we explore how biocontrol may be applied to manipulate these ecosystems to reduce the persistence of pathogens in the built environment.

### Biocontrol in the built environment

In agriculture, biocontrol has been rigorously investigated as a replacement for pesticidal, antimicrobial, and antifungal compounds used to eliminate disease-causing organisms [74, 75]. As many plant-associated microbes are naturally antagonistic towards human-associated pathogens [76], the adaptation of this method in built environments has become an emerging area of research. The *Bacillus* species used in probiotic cleaning products are also often associated with plants and soil [77]. Additionally, *Priestia megaterium* (previously *Bacillus megaterium)* is often included in these formulations due to its biocontrol activity in agriculture [78]. Some *Bacillus* species are also part of the healthy human microbiome and can confer health benefits when administered as an oral probiotic. For example, consuming *B. subtilis* spores can decolonize *S. aureus* from the human gut [79]. *Staphylococcus aureus* is an opportunistic pathogen and present in approximately one-third of the human population [80-82], suggesting that decolonization can lower the overall risk of developing future *S. aureus* infections, although we also concede that removal of beneficial *Staphylococcus* species and strains may have negative health impacts.

*Bacillus* species form spores when starved for nutrients, making them resistant to damage by heat, UV, chemicals, and desiccation. Spores remain in this dormant state until conditions improve and then germinate upon exposure to certain amino acids, such as l-alanine, l-valine, and l-asparagine [83]. This makes them ideal for inclusion in cleaning solutions because unlike probiotics that contains vegetative cells (which must be refrigerated), a spore-containing solution can be stored at room temperature indefinitely. Additionally, *Bacillus* spores will remain viable on built environment surfaces for at least 72 hours [84], can be incorporated into materials [85] where they can repair microfractures [86], and facilitate changes to a material’s shape in response to changes in humidity [87]. In the future, building materials might contain spores for the purpose of maintaining structural integrity and improving the health of occupants via biocontrol mechanisms and immune stimulation [88, 89], although research in this area is still in the very early stages.

**Figure 1:**
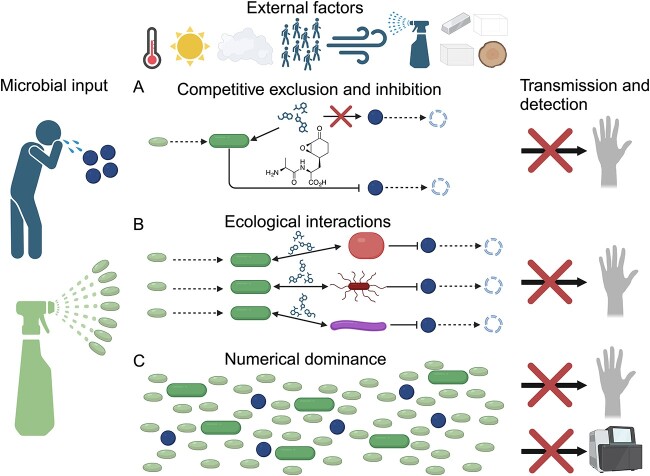
Potential pathogens can be deposited on surfaces by infected occupants, but their survival and transmission capability are dependent on many environmental factors, including temperature, light, humidity, occupant density, air flow, cleaning methods, and surface material type [43, 101-105]. However, this rate of accumulation and transmission can also be altered through manipulation of surface ecology by introducing bacteria into the environment. Here, we propose three potential modes of action for how probiotics can decrease pathogen exposure and infection risk. (A) Bacterial spores germinate and exclude pathogenic bacteria through direct consumption of resources or by inhibiting pathogen growth via the production of inhibitory molecules, such as bacilysin [106]. (B) Germinated probiotic bacteria alter microbial community interactions through metabolic exchange, leading to the inhibition of pathogens [96]. (C) Cleaner-associated bacterial cells outnumber cells of potential pathogens, resulting in numerical dominance. We hypothesize this could then reduce the rate of occupant interaction, or it may reduce the detection of pathogens by sequencing techniques.

We posit that there are two potential mechanisms by which biocontrol bacteria, such as *Bacillus* spp., may regulate pathogen exposure in built environments: (A) competitive exclusion, either through competition for nutrients or through antibiotic production and (B) enhancing the ecological stability and pathogen exclusion potential of the native surface microbiome (Fig. 1). Additionally, as outlined below, studies have shown that *Bacillus* administration in hospitals leads to a significant reduction in the abundance of known pathogens on surfaces [22-24, 84, 90, 91]. This could be due to competitive exclusion or by influencing the ecological stability of the native community; another possibility is that the application of billions of *Bacillus* spores leads to overwhelming numerical dominance (iii) that results in a decrease in the ability to detect pathogens and maybe diminished likelihood of occupant exposure due to a reduction in the probability of encountering a pathogenic cell (Fig. 1). Below we explore specific examples that may help elucidate which mechanism or mechanisms is most likely.

**Figure 2:**
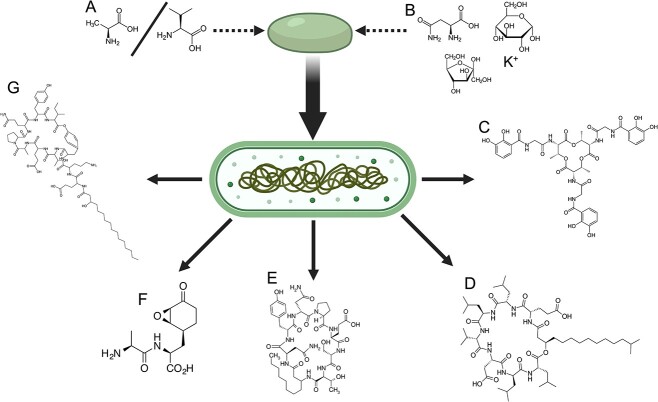
The germination of *Bacillus s*pores is triggered by either (A) *L*-alanine/*L*-valine or (B) a combination of *L*-asparagine, glucose, fructose, and potassium ions [107]. Vegetative *Bacillus* cells can generate different compounds that can exclude pathogens, including (C) the siderophore bacillibactin, which binds iron and is involved in competitive exclusion of nearby microbes [108]; (D) surfactin, which has antimicrobial properties and also aids in movement across surfaces [109]; (E) bacillomycin, which has potent antibacterial activity [110]; (F) bacilysin [106]; and (G) fengycin [111], which are both antifungal compounds.

*Bacillus* species can competitively exclude other organisms through the preferential uptake of nutrients from their surrounding environment. For example, iron, an important element for all life due to its frequent use as a cofactor in enzymes, can be sequestered by siderophores secreted by *Bacillus* species [92]. These molecules bind tightly to individual iron atoms and require specialized membrane transport proteins for uptake and utilization by a cell. Cells without the proper transportation proteins are unable to import and use the iron, thereby inhibiting their growth [93]. Competitive inhibition of pathogens can also occur via the direct production of antagonistic compounds by *Bacillus* species, including surfactin, iturin, fengycin/plipastatin, bacillomycin, and bacilysin (Fig. 2; [94]), although the effectiveness of these compounds has not specifically been demonstrated in the built environment. Several studies have demonstrated that *Bacillus* intervention enhances the pathogen exclusion properties of the extant surface microbiome [95, 96]. However, the lack of a defined mechanism of action behind such biocontrol suggests an urgent need to perform new studies aimed at elucidating these modalities. This is especially important due to the proliferation of commercial probiotic cleaners, whose efficacy should be validated against traditional cleaning methods and any impact on material integrity or human health assessed. However, studies that seek to demonstrate the effectiveness of an intervention against pathogen reduction cannot introduce pathogens into the environment to start the experiment and instead must rely on already existing exposure events, such as those occurring in hospitals. Additionally, there is a lack of control for complex factors such as occupancy rates, sunlight, and seasonality, which makes these studies susceptible to misleading conclusions (Fig. 1).

A recent study showed that it was possible to impregnate materials with a bacterial spore-containing medium, either through direct inoculation [97] or through embedding spores in 3D-printed materials [85]. Spores of genetically modified *Bacillus* have been printed into soft hydrogel materials, where they remain viable and become metabolically reactive in response to a pathogen (e.g. *S. aureus*), resulting in the biocidal activity of those agents [85]. Using sprays, materials can also be seeded with microbial spores. To test the activity of these spore sprays, a commercially available cleaner containing either *Bacillus* spp. spores or a spore-free version of the cleaner was applied to steel coupons; then, the coupons were inoculated with either *Acinetobacter baumannii* or *K. pneumoniae* [95]. The coupons were sampled at 3, 24, and 72 hours for colony-forming unit (CFU) enumeration to determine whether the presence of the cleaner, with or without *Bacillus* spores, decreased the survival rate of the pathogens compared to cleaner-free controls [95]. However, there was no significant difference in *A. baumannii* survival rates in the presence or absence of the cleaner, with or without *Bacillus*. *Klebsiella pneumoniae* survival was significantly reduced when exposed to any cleaner, and the presence of *Bacillus* did not enhance this effect. In partial explanation for this finding, metatranscriptomic sequencing data suggested a low (~1% of reads) recovery of *Bacillus* when in spore form, whereas samples with vegetative cells had ~40% of reads identified as *Bacillus*. The absence of any impact of *Bacillus* on pathogen survival, coupled with the lack of vegetative transcriptional activity, suggests that most of the spores failed to germinate and hence had no inhibitory effect on the pathogens. Another study tested the natural seeding of blocks of ceramic, linoleum, and stainless steel in both indoor and outdoor environments that were periodically cleaned over 8 months using either bleach, tap water, soap, or a *Bacillus*-containing probiotic cleaner [96]. Following this seeding period, clinical *E. coli* and *S. aureus* strains were deposited on the blocks and desiccated. Their survival was then assessed after 24 hours via CFU enumeration. Blocks cleaned with either soap or the probiotic cleaner had almost no viable pathogens after 24 hours; however, pathogen abundance on the tap water and bleach-cleaned blocks was significantly higher. Additional linoleum blocks were seeded and cleaned as before and then inoculated with a fluorescently labelled *P. aeruginosa* strain. Following a 48-hour incubation within a flow cell with tryptic soy broth, the blocks were imaged to assess the extent of surface colonization by *P. aeruginosa*. Almost no growth was observed for the blocks cleaned with tap water or soap, but there was extensive colonization by *P. aeruginosa* on the bleach and probiotic cleaner blocks. These lab-based studies shed light on how *Bacillus*, the primary biocontrol bacteria used in commercial probiotic cleaning products, behaves in different environments and varied microbial communities. However, some lab studies show that *Bacillus* mostly fails to germinate and has a limited impact on the survival of pathogens like *A. baumannii* and *K. pneumoniae* [95]. Conversely, using a probiotic cleaner appears to establish a microbial ecosystem that effectively kills or inhibits pathogens, but that cleaning with soap also leads to a microbial community that is just as effective at pathogen inhibition [96]. However, the *Bacillus* cleaner did not appear to prevent the accumulation of *P. aeruginosa* when inoculated in a rich media, suggesting that environmental conditions are important for metabolic ecology. Although each study used different methods and conditions, they together reveal that *Bacillus* species do not automatically thrive and effectively compete with other microbes on surfaces. There appear to be only specific situations where *Bacillus* can effectively compete against other microbes in the built environment, which highlights the need for detailed investigation of biocontrol microbes on varied surface types before wide-scale deployment.

An important early study on the effectiveness of biocontrol cleaning methods used a solution containing *B. subtilis*, *B. pumilus*, and *P. megaterium* spores [21] over 24 weeks. These spores reduced the CFUs recovered from coliforms, *S. aureus*, *C. difficile*, and *Candida albicans* on surfaces by 50%–89% after 3–4 weeks of application as compared to surfaces cleaned using standard cleaning methods. The observed reduction remained steady throughout the remaining 4 months of the study, but pathogen abundance rebounded when traditional cleaning was reintroduced. A subsequent study focused on the resistance of the surface microbial community to antibiotics when a probiotic cleaning solution was used, as well as whether patients were colonized by the *Bacillus* species in the cleaner and if the *Bacillus* germinated on the surfaces [84]. Spores were found to germinate on the dry, relatively nutrient-deficient surfaces but were not found in samples of blood and urine from patients. Again, a decrease in pathogen abundance was observed on surfaces, as well as a reduction in the abundance of genes associated with antibiotic resistance. The study found no evidence that the *Bacillus* spp. were acquiring antibiotic resistance genes from the native microbial community.

These results prompted a large multicentre study focused on investigating whether *Bacillus*-based cleaning products could reduce the resistome of hospital-associated pathogens [98]. A previous study demonstrated that healthcare-associated pathogens tended to acquire additional antibiotic resistance and virulence genes over time in the hospital [25]. The internal medicine wards of five Italian hospitals were enrolled and samples were collected for an initial 6-month period, during which traditional cleaning methods were continued. Following this baseline period, a 6-month intervention was carried out using a Probiotic Cleaning Hygiene System (PCHS) that consisted of *B. subtilis*, *B. pumilus*, and *P. megaterium* spores, after which there was a 6-month postintervention period. The use of PCHS was associated with an up to 99% reduction in AMR genes contained in the hospital microbiome, with a 33%–100% decrease in the presence of resistant strains. The use of PCHS was also associated with a 60% decrease in the consumption of antimicrobial drugs by patients, leading to a 75% reduction in the costs associated with treating AMR infections. The cumulative incidence of healthcare-associated infections decreased significantly in the post-PCHS treatment period compared to the conventional treatment phase, from 4.8% to 2.3% (*p* < .0001). These results are promising, but replication by other research teams is necessary to increase confidence in this approach, as well as further investigations into mechanisms of action. Lacking this knowledge severely limits our ability to refine the process and to understand the ecological dynamics that underpin the observations.

Additional follow-up studies have confirmed the effectiveness of probiotic surface treatments in reducing the abundance of AMR genes and the potential pathogens that often harbour them. For example, bacteriophages are an attractive fast-acting alternative to spores. In a study using the PCHS combined with phage targeting *Staphylococcus* in hospital bathrooms, an 87% reduction in *Staphylococcus* species (including nonpathogenic strains) was observed after only 1 day of phage treatment [90]. After six additional days, there was a 97% reduction in *Staphylococcus* observed, but there was a return to preintervention levels after 4 days of treatment cessation. Resuming the treatment for 7 days resulted in a reduction similar to that observed in the first treatment period. A larger follow-up study at two hospitals using the combined PCHS+Phage treatment had mixed results. One hospital showed a significant reduction in *Staphylococcus*, but the other did not [24]. This study was performed during the COVID-19 pandemic, which led to the use of emergency applications of 3% NaClO disinfectant. One hospital used a greater number of NaClO applications, and this increase was correlated with an order of magnitude reduction in *Bacillus* and phage abundance, likely leading to a reduction in the effectiveness of the probiotic intervention [24]. These results support an earlier study, also performed during the COVID-19 pandemic, that found a reduction in pathogen load through the use of PCHS was reversed when emergency 5% NaClO was implemented [91]. These recent studies highlight the importance of understanding the dynamic nature of real-world applications, the limits of using phage as a treatment, and how probiotic cleaning methods might be rendered ineffective when harsh bleach cleaning agents are used in tandem.

## Conclusions and future directions

Probiotic intervention studies in hospitals [22-24, 99, 100] have demonstrated the potential to revolutionize cleaning approaches in healthcare facilities. However, much is still unknown about their mechanism of action, as well as how additional factors could influence their efficaciousness *in situ*. Further investigation of how spores germinate and competitively exclude other microbes in these dry, nutrient-depleted environments is needed for the successful development and broad deployment of probiotic cleaners. To answer these questions, we must use a variety of experimental strategies, including varied application methods (i.e. how the cleaner is applied and at what concentration is the cleaner most effective), inclusion of taxonomically diverse pathogenic microbes at varied concentrations, and a characterization of whole community dynamics in response to cleaner application. These experiments will require highly controlled and easily manipulatable conditions, and therefore, laboratory testing will be essential for determining the relative importance of factors associated with method of use, humidity, nutrient availability, surface material type, and cleaning regimes. Further, simulating transmission events of microbes from surfaces to humans will be needed to more fully understand how probiotic cleaners influence transmission rates of taxa of interest.

Differential use patterns of healthcare facilities might alter best practices for probiotic cleaner use. For example, it is unknown how the magnitude of response might vary between different wards or rooms. Patient rooms and bathrooms have been the primary focus of intervention studies; however, other shared spaces, such as hallways or visitor areas, also carry the potential for pathogen accumulation and transmission risk. Additionally, long-term monitoring is crucial to detect whether the pathogens in these environments begin to adapt to this new cleaning method and therefore become resistant to biocontrol. Such a situation could start an evolutionary “arms race” with unknown outcomes. It is crucial to consider the health and emotional impacts on the patients and staff. Sociological studies on healthcare workers and patients are necessary to understand the perception that patients, staff, and the public have about the use of these novel products and to involve their feedback in product design and application. The popularity of probiotics in popular foods and drinks could help alleviate concerns, but deploying them in hospitals, especially around high-risk patients, will likely be controversial. Understanding the perception of these products, and explaining how and why they work, will aid in successful widespread adoption across healthcare systems. As seen in studies conducted during the COVID-19 pandemic, harsh cleaning treatments using bleach may inhibit the probiotic and phage cleaners, so it will be imperative that staff are trained in how to properly treat surfaces cleaned with probiotics.

Beyond direct application to the engineering of probiotic materials [85], 3D printing is primed to be the future of microbial biocontrol in built environments. Recent advances in 3D printing have resulted in materials that can be printed at low enough temperatures that spores and even live bacteria are not harmed during the assembly process. Unlike previous efforts, which used hydrogels [85], these new approaches can enable the printing of ceramics or hard plastics with improved utility as building materials. Using genome-enabled metabolic modelling to select bacteria that are optimized for these printing processes presents unique opportunities for built environment-specific designs that could be used to manipulate the ecology of these environments in prescribed ways. If this trend continues, we may soon be able to print tiles, furniture, or even entire structures out of sustainably produced microbially active materials [97]. Establishing the efficacy of these products through well-designed field intervention studies that are informed by lab-based experiments is an exciting subfield of built environment microbiome research and will ultimately lead to better products, building materials, and design techniques in buildings to improve occupant health and well-being.

## Data Availability

Data sharing not applicable to this article as no datasets were generated or analysed during the current study.
